# Volatile Fatty Acids in Ruminal Fluid Can Be Used to Predict Methane Yield of Dairy Cows

**DOI:** 10.3390/ani9121006

**Published:** 2019-11-20

**Authors:** S. Richard O. Williams, Murray. C. Hannah, Joe L. Jacobs, William J. Wales, Peter J. Moate

**Affiliations:** Agriculture Victoria Research, Ellinbank, VIC 3821, Australia; murray.hannah@agriculture.vic.gov.au (M.C.H.); joe.jacobs@agriculture.vic.gov.au (J.L.J.); bill.wales@agriculture.vic.gov.au (W.J.W.); peter.moate@agriculture.vic.gov.au (P.J.M.)

**Keywords:** methane yield, proxy, ruminant, cattle, dairy, beef

## Abstract

**Simple Summary:**

Methane emissions from cattle are difficult to measure, and some proxies used to estimate them have required information that is not always available. An example is predicting methane from the fatty-acid profile of milk, but this strategy is not suitable for non-lactating animals. We propose equations to predict the methane emitted per unit of feed eaten (methane yield) based on the volatile fatty acids within the rumen of the animals. Three of the seven equations we investigated were equally good at predicting the methane yield of dairy cows. Validation of these equations using previously published results indicated that the equations should also work for beef cattle. Being able to predict the methane yield for all classes of cattle means that a single strategy can be used, eliminating differences because of the use of different methods for different animal classes. Further work is necessary, but our strategy should be able to be adapted for use in cattle production environments. Being able to predict the methane of production animals will enable accurate estimates of the methane emissions from those animals, and assessment of strategies to reduce those emissions.

**Abstract:**

The dry matter intake (DMI) of forage-fed cattle can be used to predict their methane emissions. However, many cattle are fed concentrate-rich diets that decrease their methane yield. A range of equations predicting methane yield exist, but most use information that is generally unavailable when animals are fed in groups or grazing. The aim of this research was to develop equations based on proportions of ruminal volatile-fatty-acids to predict methane yield of dairy cows fed forage-dominant as well as concentrate-rich diets. Data were collated from seven experiments with a total of 24 treatments, from 215 cows. Forage in the diets ranged from 440 to 1000 g/kg. Methane was measured either by open-circuit respiration chambers or a sulfur hexafluoride (SF_6_) technique. In all experiments, ruminal fluid was collected via the mouth approximately four hours after the start of feeding. Seven prediction equations were tested. Methane yield (MY) was equally best predicted by the following equations: MY = 4.08 × (acetate/propionate) + 7.05; MY = 3.28 × (acetate + butyrate)/propionate + 7.6; MY = 316/propionate + 4.4. These equations were validated against independent published data from both dairy and beef cattle consuming a wide range of diets. A concordance of 0.62 suggests these equations may be applicable for predicting methane yield from all cattle and not just dairy cows, with root mean-square error of prediction of 3.0 g CH_4_/kg dry matter intake.

## 1. Introduction

Mitigation of enteric methane from ruminants requires methods to estimate the extent of mitigation. Many methods rely on complex equipment and skilled people, and while they are suitable for research purposes, they are unsuitable for routine use on farms [1,2]. An alternative has been to use proxies of methane production (g/d) such as dry matter intake (DMI) and milk fatty acids [3].

The total DMI of cattle fed high-forage diets can be used to accurately predict their methane emissions [4]. However, many animals are fed enriched diets in which concentrate constitutes more than 300 g/kg of the diet [5,6] and these diets decrease the methane yield (g/kg DMI) of those animals [7]. A range of equations to predict methane production (g/day, 34 equations) and methane yield, (7 equations) were compared by Niu et al. [8]. These equations were based on various combinations of DMI and chemical descriptors of the consumed feed (e.g., concentrations of neutral detergent fiber and ether extract) as well as animal descriptors such as bodyweight, milk yield, and milk fat concentration. Niu et al. [8] concluded that the more complex equations were better than a simple equation based on DMI alone. The more complex approach may be suitable for situations where the feed intake of individual animals can be accurately measured and where the composition of the feed consumed by each individual animal is known. However, such an approach is unlikely to apply where DMI is unknown or difficult to measure, such as animals fed in groups or grazing.

Milk fatty acids are an alternate proxy to predict methane production and methane yield [9,10]. However, measurement of long-chain fatty acids in milk by gas chromatography is relatively expensive and the approach has been found to be inaccurate at predicting methane emissions and yield if cows are fed diverse diets [11].

Production of volatile fatty acids (VFA) in ruminal fluid, including acetate, propionate, and total butyrate (n-butyrate plus iso-butyrate) have been related to methane production using stoichiometric equations [12,13]. In addition, changes in the acetate to propionate ratio because of treatment effects have been associated with changes in methane production both in vitro [14] and in vivo [15,16], while changes in (acetate + butyrate)/propionate have also been associated with changes in methane production in vivo [13,17]. While no correlations were reported between ruminal VFA proportions and methane yield, the associations suggest that VFA proportions in ruminal fluid could be used as a simple proxy to predict methane yield of dairy cows.

In previous work, researchers have noted associations between VFA proportions in ruminal fluid and methane production within their single experiment [18,19], but none have presented equations to predict methane yield from the proportions of volatile fatty acids in ruminal fluid. Therefore, the aim of our research was to use a database of methane yield and proportions of specific VFA in ruminal fluid to develop and validate equations for their accuracy in predicting methane yield of dairy cows.

We hypothesized that the methane yield of dairy cows offered a wide range of diets could be predicted from the proportions of VFA (mol/100 mol total VFA) in ruminal fluid.

## 2. Materials and Methods 

In all collated experiments, animals were cared for according to the Australian Code of Practice for the Care and Use of Animals for Scientific Purposes [20,21]. Animal use was approved by the Agricultural Research & Extension Animal Ethics Committee of the Department of Jobs, Precincts and Regions–Victoria (Approvals 2009-31, 16 December 2009; 2010-16, 23 August 2010; 2011-24, 06 December 2011; 2012-15, 21 August 2012; 2012-27, 13 December 2012; 2013-22, 16 January 2014; 2014-12, 23 September 2014).

### 2.1. Model Development 

Models were developed using theoretical stoichiometry and previously published empirical associations. The abbreviations used in the models are listed in Table 1 with a brief description and their units.

A stoichiometric equation from Moss et al. [13] served as a basis for the development of equations to predict the methane yield of dairy cows. Equation (1a) is as presented by Moss et al. [13], with methane production designated by CH_4_P, and the substrates acetate by C2, propionate by C3, and butyrate by C4:

CH_4_P = 0.50C2 − 0.25C3 + 0.50C4, (1a)

Note that in Equation (1a) CH_4_P, C2, C3, and C4 have units of moles. However, individual VFA are often reported as moles per 100 moles of total VFA (also designated as M%), and total VFA is often reported as a concentration (mol/L). Therefore, it is necessary to do a conversion to take the units for each parameter into account. If we divide both sides of Equation (1a) by 100 moles of total VFA, then we can rewrite Equation (1a) as:

CH_4_Y = 0.50A − 0.25P + 0.50B, (1b)

where CH_4_Y is the yield of methane per 100 moles of total VFA in ruminal fluid, and A refers to acetate, P to propionate, and B to total butyrate (i.e., n-butyrate plus iso-butyrate), and these are expressed as moles per 100 moles of total VFA. If it is assumed that each kg DM consumed is fermented in the rumen to produce c moles of total VFA, then it follows that Equation (1b) can be expressed as:

MY = 16 × (0.50A − 0.25P + 0.50 B) × c/100,(1c)

where MY is defined as methane yield (g CH_4_/kg DMI), 16 refers to the molecular mass of methane (16 g/mole), and c has units of moles of total VFA/kg DMI. An inherent assumption in Equation (1c) is that A, P, and B at the time of sampling reflect the daily production rates of these VFA. However, the concentrations of VFA in a sample of ruminal fluid reflect the net accumulation of fatty acids because of the production and removal of these fatty acids from the rumen by the bulk flow of fluid from the rumen and by absorption of VFA across the ruminal wall into the blood. Each VFA have different molecular weights and are absorbed across the ruminal wall at different rates [22]. Therefore, to take account of these issues, we have examined additional models that are based on various combinations of A, B, and P and in which the units for MY, A, B, and P are the same as those in Equation (1c). In particular, to account for possible differences in net accumulation of A, B, and P within the rumen, we propose Equation (2).

MY = dA − eP + fB,(2)

Equations (1c) and (2) are based purely on stoichiometry, while the following equations follow a more empirical approach. Equation (3) is based on a commonly noted association between methane production and A/P [15,16].

MY = g(A/P) + h,(3)

Equation (4) is based on a commonly noted association between methane production and (A + B)/P [13,17].

MY = i(A + B)/P + j,(4)

Methane production has been reported to be positively associated with the molar concentration of propionate in ruminal fluid [23]. However, it is usually suggested that the association is negative [13] as the production of methane and propionate compete for metabolic hydrogen. Accordingly, Equation (5) examines the relationship between MY and P.

MY = kP + m,(5)

Equation (6) is similar to Equation (5), but [P] refers to the concentration (mM/L) of propionate in the ruminal fluid.

MY = n[P] + q,(6)

Equation (7) is a simplification of Equations (3) and (4).

MY = s/P + t,(7)

For Equations (2)–(7), the coefficients d, e, f, k, and q have units of g CH_4_·kg DMI^−1^·moles^−1^·100 moles total VFA, while the coefficients g, and i have units of g CH_4_·kg DMI^−1^, and the coefficient m has units of g CH_4_·kg DMI^−1^·L·mmol^−1^. The constants h, j, l, n, and s have the units of g CH_4_·kg DMI^−1^. All of the above coefficients and constants are determined by regression as described in the statistical analysis section.

### 2.2. Data

Individual animal data used to develop the models were collated from seven previously conducted experiments (Table 2). 

There were 215 records of methane yield with corresponding VFA proportions. Methane production was measured either by open-circuit respiration chambers over two days [32] or a sulfur hexafluoride (SF_6_) technique over three to five days [33]. Methane yields of individual animals ranged from 6.9 to 32.4 g/kg DMI (Table 3). Methane yield was positively linearly related to proportions of acetate (r = 0.557) and butyrate (r = 0.642), and positively linearly related to the reciprocal of the proportion of propionate (r = 0.745). In each experiment, ruminal fluid was collected via the mouth on one occasion at the conclusion of the methane measurement period, approximately four hours after the start of the morning feeding. Samples were collected using an oro-ruminal sampling probe, similar to the one described by Geishauser [34], and a vacuum pump [35]. Concentrations of total VFA (mol/L) and of the major individual VFA (acetic, propionic, n-butyric, and iso-butyric; mol/100 mol of total VFA) in ruminal fluid were measured by gas chromatography [35].

Additional individual-cow data for external validation were taken from an experiment of similar design involving 32 Holstein cows [6] (Table 4). Further data for validation came from 67 dietary treatment means published in the scientific literature (Table 4). These data from the scientific literature included data from both dairy cows and beef animals consuming a wide range of diets. Only articles with methane production and DMI or methane yield, and individual VFA proportions where methane was measured in calorimeters were selected. Dietary treatments containing nitrate, sulphate, 3-nitrooxypropanol, or halogenated methane analogues were not used in the validation data since they can cause changes in the methane yield with little effect on ruminal VFA.

### 2.3. Statistical Analysis and Model Evaluation

Data from individual experiments in Table 2 and their pooled data were plotted and visually inspected for linearity or curvature between methane yield and proportions of acetate, propionate, butyrate, reciprocal of propionate and acetate to propionate ratio.

Equations (1)–(7) were modelled statistically in Genstat 18 (VSN International Ltd., Hemel Hempstead, UK) using a leave-one-experiment-out cross-validation process [52,53]. All the individual cow data from one experiment were put aside as a validation set and the remaining data were used to construct a linear mixed-effects meta-analysis, with fixed-effects given by Equations (1)–(7) above. All meta-analysis models had random effects for experiment, treatment within experiment, and animal within treatment. The estimated fixed effects from each model were then used to predict methane yield data from the omitted experiment using its VFA proportions data. In this way, each experiment was used in turn as validation data, thus providing a complete set of predicted methane yields for the seven experiments for each model. These predicted methane yields were compared to the measured methane yields using the root-mean-square-error of prediction (RMSEP) calculated to assess the performance of each model:

$$\mathrm{RMSEP}\;=\;\sqrt{\frac{1}{n}\sum_{i=1}^{n}{\left(y_{i}-{ŷ}_{i}\right)}^{2}}$$

where y_i_ are observed validation data and ŷ_i_ are model predictions calculated independently of the validation data.

The root-mean-square-error of prediction can be interpreted as an average standard deviation of prediction. Lin’s concordance correlation coefficient (CCC) was also used as a measure of agreement between predicted and measured methane yield for each model [54]. The models were ranked according to their cross-validation RMSEP and CCC.

Each model was also fitted to the pooled training data of Table 2 using the mixed-effects meta-analysis model described above, to obtain a final calibrated equation for use in prediction beyond these data. The best ranked equations were validated using external data (Table 4). The methane yields of the external data were predicted by applying the equations, calibrated on the pooled training data to the VFA proportions reported in the external data. These predictions were compared to their matching reported methane yields using the measures of agreement, RMSEP and CCC. The square of RMSEP (MSEP) was decomposed into three components, these being the error-of-central-tendency, error-due-to-regression, and error-of-disturbance [55] also known as the squared-bias, non-unity-of-slope, and lack-of-correlation [56], respectively.

## 3. Results

**Figure 1:**
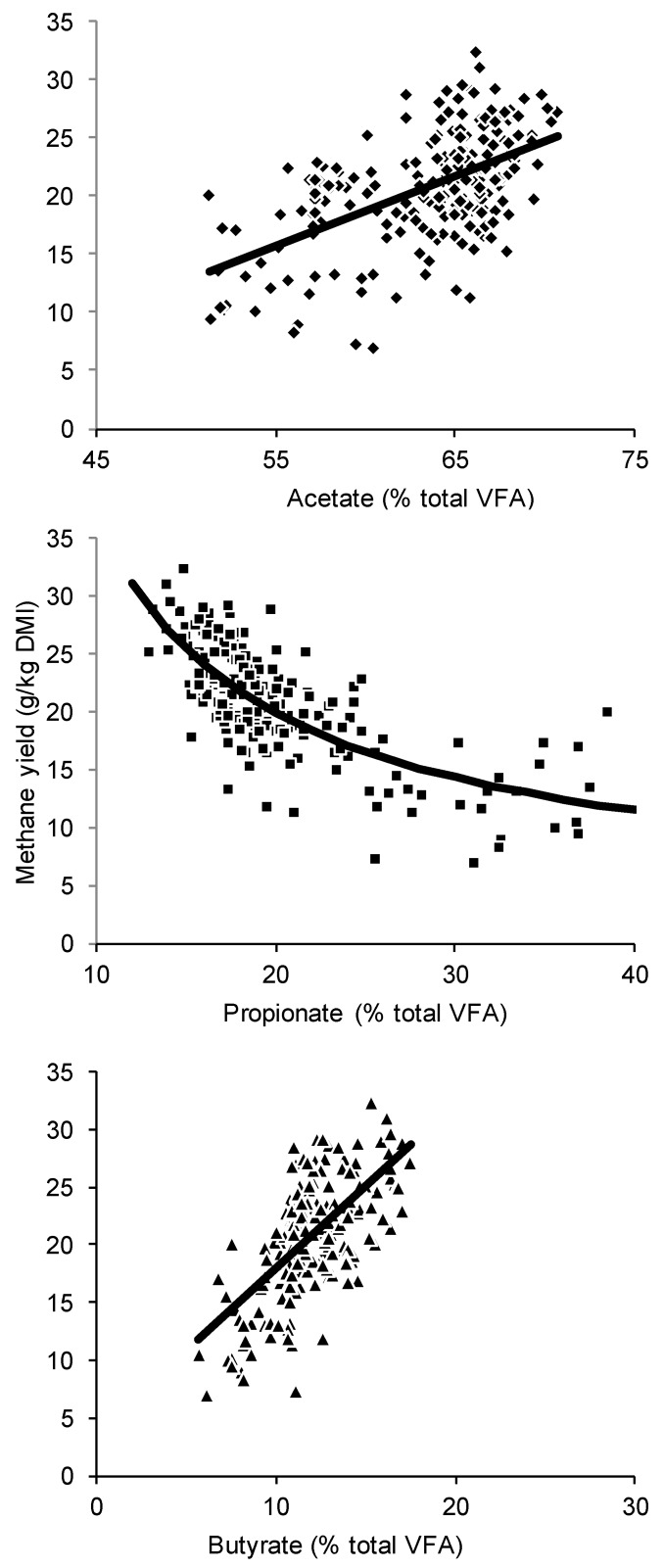
Methane yield (g/kg DMI) versus ruminal acetate, propionate and butyrate as mol per 100 mol of total volatile fatty acids from 215 cow records across 24 diets [24,25,26,27,28,29,30]. Solid lines are linear or reciprocal trend lines.

Visual inspection of the correlations between methane yield and proportions of acetate, propionate and butyrate in the pooled data showed that methane yield was positively linearly related to the proportions of acetate and butyrate, and the reciprocal of propionate (Figure 1). Within experiments, methane yield was always positively linearly related to proportions of acetate and butyrate, and was always related to the reciprocal of propionate proportions. In some experiments, and certainly for the pooled data, the relationship between methane yield and propionate proportion did not appear to be linear, whereas the relationship between methane yield and the reciprocal of propionate proportion did appear to be linear (data not shown).

Most models had similar performance on the training data in terms of their cross-validated prediction error and concordance. The exception was Model 6, based on the concentration of propionate, which had RMSEP much greater than all other models (Table 5). The best three models were Model 3, Model 4, and Model 7. These models all had the same RMSEP of 3.2 g/kg DMI. While Model 3 had a slightly lower concordance (0.69) compared to Models 4 and 7 (0.70). 

**Figure 2:**
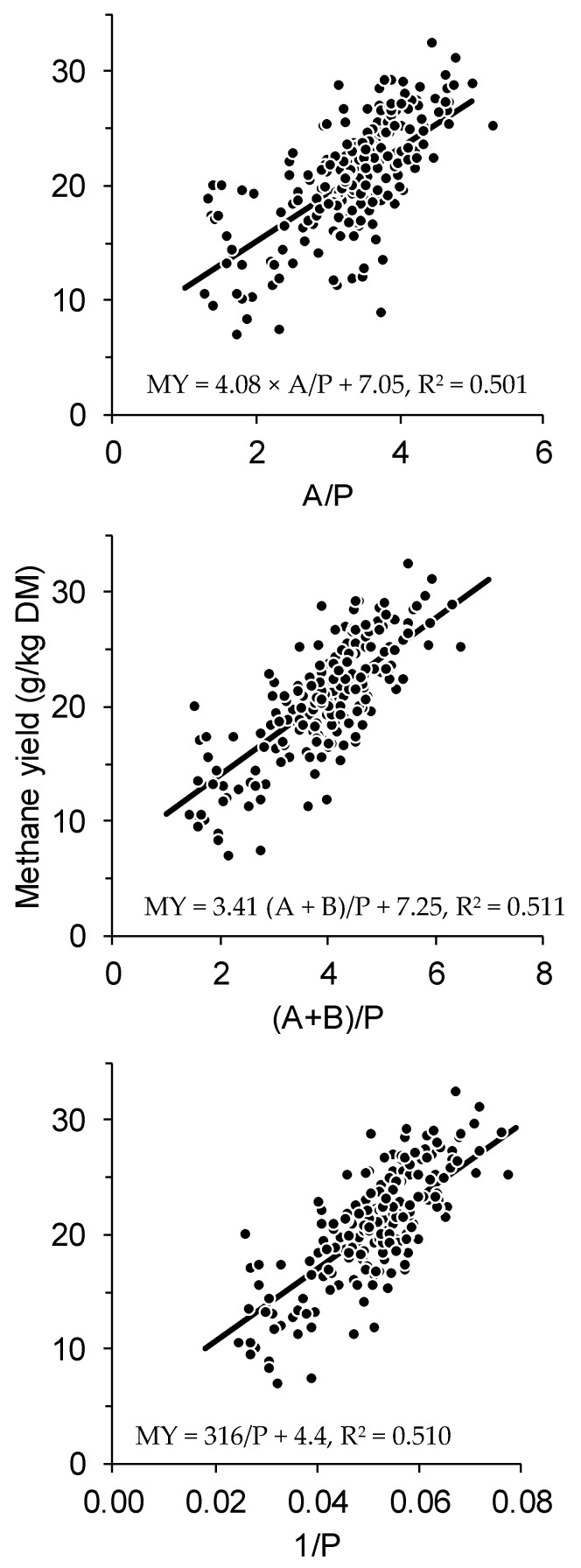
Combined methane yield (MY, g/kg DM) data from seven previously conducted experiments [24,25,26,27,28,29,30] plotted against ruminal fatty acid ratio A/P, (A + B)/P, and 1/P, with linear equations fitted by linear mixed effects meta-analysis (solid line).

The symbolic forms of all models are shown in Table 5 alongside the coefficient estimates for these models, fitted to the pooled training data of all seven experiments. The equation derived from stoichiometry (Equation 1c, Table 5) was the second poorest model, in terms of having the second greatest RMSEP. Ranking of the concordance between measured and predicted methane yield for the models tested reflected the ranking of the RMSEP. The equations for Models 3, 4, and 7 are shown against the combined training data in Figure 2. Components of variance for deviations were 1.7 for experiment, 1.5 for treatment within experiment, and 6.9 for cow, for Model 4 (similar for Models 3 and 7). Each component was statistically significant (*p* < 0.05).

**Figure 3:**
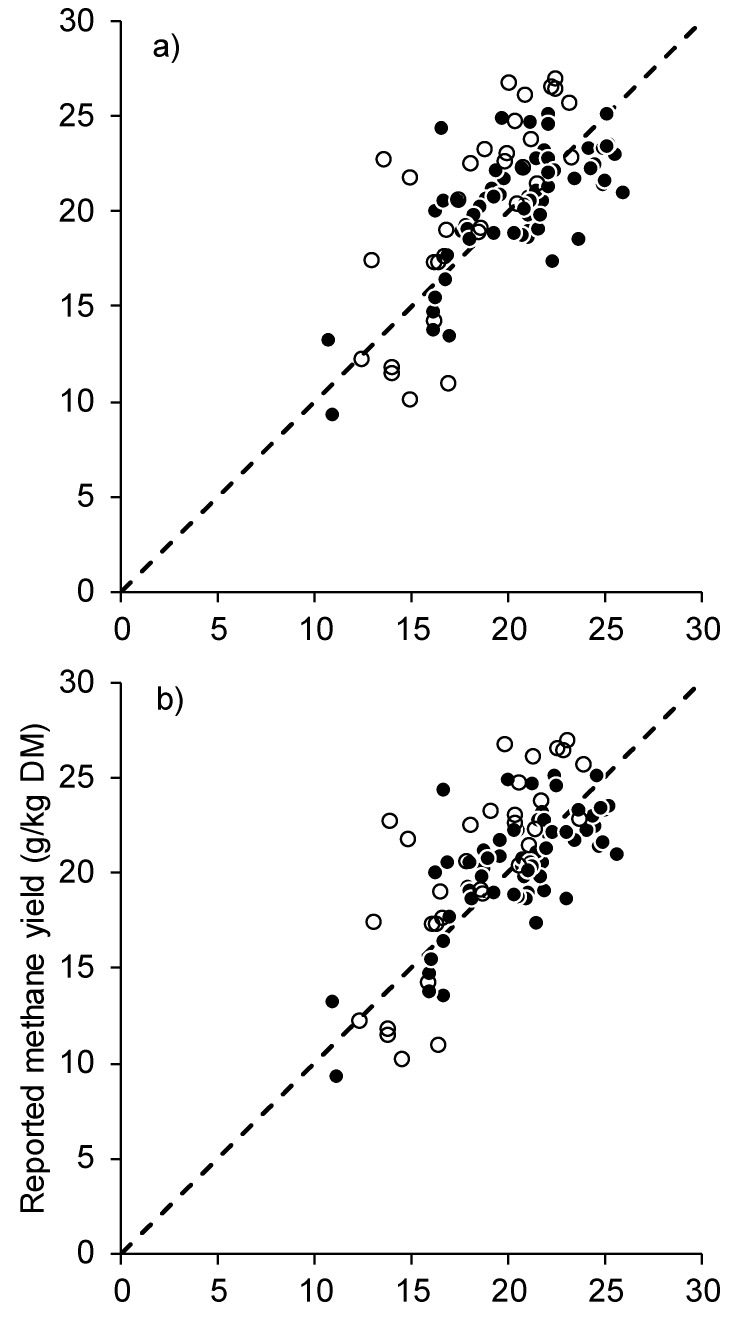

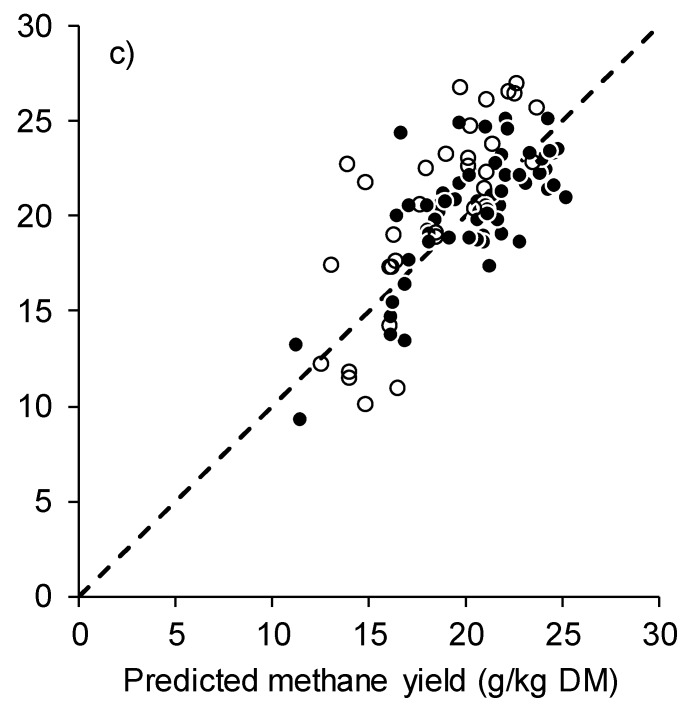
Independent validation of (**a**) Equation (3), (**b**) Equation (4), and (**c**) Equation (7) showing the observed methane yield against equation-predicted methane yield, for 32 individual [6], (open circles) and 67 treatment means from 16 previously published experiments [14,36,37,38,39,40,41,42,43,44,45,46,47,48,49,50,51] (closed circles) with 1:1 line of agreement (dashed).

The RMSEP between the observed methane yield of individual cows in unrelated datasets [6] and the methane yield predicted by Equation (4) was 3.62 g/kg DMI and concordance correlation coefficient was 0.66. The components of MSEP were 3.3 for error-of-central-tendency, 0.47 for error-due-to-regression, 9.3 for error-of-disturbance. When group means from published experiments (Table 4) were used, the RMSEP was 2.99 g/kg DM and concordance was 0.62 (Figure 3). The components of MSEP were 0.62 for error-of-central-tendency, 0.20 for error-due-to-regression, and 8.1 for error-of-disturbance. Similar results were obtained for Equations (3) and (7) (data not shown).

## 4. Discussion

Methane yield of dairy cows can be predicted from the proportions of VFA in their ruminal fluid, confirming our hypothesis, but the concordance between observed and predicted methane yield differed between the seven models tested. Equation (3) is consistent with previous research that reported a proportional relationship between methane yield and acetate to propionate ratio [15,18,57]. However, there are also reports of no relationship [37,45,51]. The reason for this is not clear but may relate to the different dietary treatments used, and this is discussed later as a limitation of our prediction models. Equation (4) reflects previous reports of methane yield being proportional to (A + B)/P [17,19]. The relationship between methane yield and (A + B)/P was consistent both within and between the 7 experiments in our study, notwithstanding significant variation associated with experiment, treatments, and animals around the average line. Summing the components of variance estimates for experiment and treatment imply a lack-of-fit standard deviation of 1.8 g CH_4_/kg DMI associated with feed-type and context, with the remaining error (SD =2.6 g CH_4_/kg DMI) being due to animal or measurement. Equation (7) supports the contention that the production of methane and propionate are in competition [58] but no reports linking methane yield solely with the reciprocal of propionate proportion were found. Propionate proportion alone (Equation 5) was not a good linear predictor of methane yield, which is to be expected given that the data suggested a reciprocal relationship between methane yield and propionate proportion. Despite this, in a previous study on 532 Angus bulls (1 to 2 years age) fed a diet containing lucerne and oaten hay chaff, a significant positive correlation between methane yield and propionate proportion was reported [59]. However, in the work of Herd et al. [59], feed was offered once per day and ruminal fluid was sampled 24 h after the start of feeding compared to the twice per day feeding and sampling 4 h after the morning feed used in our investigations. Subsequent work by Smith et al. [60], in which ruminal fluid was sampled 3 h after the start of feeding, reported a weak negative correlation between methane yield and individual VFA proportions.

Validation of our best equations showed there was a strong concordance between the observed and predicted methane yield. The experiment of Moate et al. [6] used the same sampling and measurement processes as those experiments used to develop the equations, so strong concordance was expected. Despite the literature data being from conditions different to ours (sampling, diets, and animals), the concordance between the mean methane yield reported in the scientific literature and the methane yield as predicted by Equations (3), (4) and (7) was greater than 0.60. The components of MSEP show that the lack of agreement between the independently observed and predicted methane yields was dominated by error of disturbance, that is, random error rather than either mean or slope bias. This suggests that our best equations are robust and using them to predict methane yield from ruminal VFA proportions may be applicable for predicting methane yield from all cattle and not just dairy cows. Thus, our equations offer a low-cost approach to screen and rank large numbers of animals for methane yield.

Equations derived from stoichiometry can be used to predict the quantity of methane produced from the quantity of individual VFA in the rumen, but caution is required when applying them. It must be remembered that Equation (1a) predicts the quantity of methane from a quantity of ruminal VFA and as such has applicability at the one instant when the ruminal VFA are sampled. Even after translating Equation (1a) into terms of methane yield (Equation 1c), its performance was inferior to five of the other six models. Other research in this area has focused on using feed intake and feed composition to predict ruminal VFA production and then using stoichiometry (Equation 1a) to predict methane production from predicted VFA production [61]. Despite the sophistication of the model by Alemu et al. [61], it had poor correlations between measured and predicted VFA concentrations and hence the model of Alemu et al. [61] had poor accuracy at predicting methane production. It is important to note that individual VFA may be absorbed from the rumen at different rates [22]. A consequence of this is that proportions of individual VFA in ruminal fluid may not reflect the proportions at which they were produced, which may explain why Equation (1c) had a large RMSEP. What remains unclear is why Equation (2) performed only slightly better than Equation (1c), given that adjusting the coefficients should have taken into account any difference between proportions of individual VFA produced and their proportions in ruminal fluid. 

Using the proportions of total butyrate instead of *n*-butyrate in our prediction equations had no appreciable effect on the predicted methane yield. It was expected that *n*-butyrate might be a better parameter than total butyrate to predict methane yield. This is because iso-butyrate is not synthesized by ruminal bacteria, but is derived from deamination of branched chain amino acids and its formation does not provide protons for methane production [62]. Preliminary testing of our models using either proportions of total butyrate or *n*-butyrate showed there were only minor differences in the prediction equations and no significant differences in the predicted methane yields (data not shown). This outcome was probably due to *n*-butyrate being 93% of the total butyrate in our development data set. We also note that while the production of iso-butyrate may not directly result in the production of protons that can be utilized to produce methane, iso-butyrate has been reported to stimulate ruminal cellulose digestion [63] and we speculate that this indirect mechanism may lead to greater methane production. Given there was no appreciable difference in the accuracy of prediction whether we used total butyrate or *n*-butyrate and that many scientific publications have reported butyrate without qualification as to type, we opted to use total butyrate in our prediction equations so they could be used to predict methane yields on data from any experiment.

Our yield of total VFA per kilogram of DMI was similar to previous work. An early experiment used a combination of in vitro and in vivo methods to estimate the production rate of total VFA [64]. Using data reported by Stewart et al. [64] we calculate that their yield of total VFA was, depending on the diet, between 3.0 and 3.3 mol total VFA/kg DMI. In the study by Stewart et al. [64] there were several unverifiable assumptions that were made regarding the production rates of total VFA and they admit that their “production figures presented earlier should be considered as minimal.” More recently, Lee et al. [65] fermented dried grass in a RUSITEC system and reported a yield of 3.87 mol of total VFA/kg DMI, a value that is much closer to our estimate. Overall, we consider our estimate of 3.98 mol total VFA/kg DMI to be plausible.

The variability in the observed relationships between methane yield and ruminal VFA proportions may be due to activation of different fermentation pathways, measurement error, or the sampling protocols. In addition to the fermentation pathways proposed by Moss et al. [13], there are others as outlined by Bryant [66], and these may come into effect differently when cows consume a wide range of diets or have diverse ruminal microbial populations [8]. We speculate that these diverse fermentation pathways might alter the relationship between ruminal VFA proportions and methane production. Measurement error is present regardless of the technique used to estimate methane yield [67]. All equipment used by us was calibrated prior to use and validated after use, and our SF_6_ technique has been shown to be comparable to respiration chambers [33]. However, there is still variation because of the factors that cannot be accounted for, such as the extent of the effect that the previous day’s DMI has on today’s methane emission [68]. Sampling protocol, particularly the duration between feeding and sampling of ruminal fluid has been shown to affect the proportions of VFA in ruminal fluid and hence influence the strength of the correlation between methane yield and proportion of VFA [23,45]. While there are differences in sampling periods and in the intervals between feeding and sampling of ruminal fluid there will always be unaccounted for variation between methane yield and individual VFA proportions in ruminal fluid. Alternatively, if multiple samples of ruminal fluid were collected from individual cows over the same period as the methane measurement, then we speculate that mean proportions of specific VFA applied to the models tested in this research would result in a dataset and equations with greater correlations to methane yield and smaller errors of prediction than those which we have reported. However, this would require further research. If precision is not required, for example if animals are only being ranked, then our best equations may be suitable without further improvement.

Predictors of, or proxies for, methane yield have tended to focus on DMI and diet composition [69]. Although such models have had some success, it seems unlikely that they can take account of ruminal microbes adapting over time to a particular diet and this could cause considerable errors in prediction [70]. Furthermore, prediction equations based on DMI and diet composition may not be applicable to grazing situations where it is difficult to accurately estimate DMI or the chemical composition of what animals actually select from a mixed pasture [71]. Milk has also been investigated as a predictor of methane yield. Dijkstra et al. [10] developed an equation to predict methane yield from milk fatty acids (R^2^ = 0.73, RMSEP not reported, concordance not reported) using 50 observations covering 10 diets. Furthermore, Niu et al. [8] used a database of over 3000 records and reported several equations to predict methane yield from feed and milk characteristics. The best equation of Niu et al. [8] used concentrations of dietary fat and neutral detergent fiber, energy corrected milk yield, milk fat concentration, milk protein concentration, and body weight (RMSEP = 14.7% of mean, concordance 0.37). However, for grazing cows the detailed composition of feed eaten is seldom known. When Niu et al. [8] used a simpler model based on energy corrected milk yield and milk composition, it resulted in similar predictive power to their more complicated equation (RMSEP = 15.1% of mean, concordance = 0.30). In comparison, our Equation (4) had R^2^ = 0.60, RMSEP = 15.4% of mean observed methane yield and a concordance of 0.73 between predicted and observed methane yield. This error is similar to that of Niu et al. [8] but the concordance of our equation is much greater. In addition, our equation does not require any knowledge of diet and can be used in non-lactating animals. Sampling ease and analysis cost must also be taken into consideration. Milk samples are easy to obtain but currently more expensive to analyze for fatty acids compared to the cost of analyzing ruminal fluid for VFA. The collection of ruminal fluid is a common procedure in many nutrition related experiments on ruminants to enable the measurement of a range of parameters in ruminal fluid. Thus, the additional measurement of VFA in ruminal fluid is not necessarily onerous, but would require the use of a standard protocol and skilled technicians. 

Using VFA proportions to predict methane production as distinct from methane yield, has not been very successful to date. Propionate concentration has been correlated with methane production of 12 Merino-cross ewes, but explained only 26% of the variation in methane production [23]. Robinson et al. [23] concluded that “none of the suite of VFA parameters assessed offers a useful tool to predict methane production in grazing sheep.” More recently, a review on this topic concluded the relationships between methane production and ruminal VFA were variable and not as straight forward as theory suggested [3]. These pessimistic conclusions regarding the prediction of methane production from ruminants are only partly warranted. The DMI of individual animals may vary greatly, and DMI is the principal determinant of methane production in ruminants [4,72]. If DMI of an individual animal can be measured or accurately estimated and since methane yield can be predicted from VFA proportions in ruminal fluid, then methane production can be estimated as the product of DMI and methane yield. Therefore, contrary to the conclusions of Robinson et al. [23] and Negussie et al. [3], we consider that VFA parameters in ruminal fluid, when combined with measured or estimated DMI, do offer a potential tool to predict methane production in ruminants. 

There are limitations when using ruminal VFA to predict methane yield or methane production. In comparison to control diets, diets that contain either nitrate, sulphate, 3-nitrooxypropanol, or halogenated methane analogues can cause substantial reductions in methane yield and methane production with little or no effect on ruminal VFA proportions [47,73,74]. For example, while Equation (4) can predict the methane yield of control diets with errors less than 9%, the prediction errors associated with diets containing sulphate were 21%, nitrate 40%, nitrate plus sulphate 85%, and 3-nitrooxypropanol 67%. Similar prediction errors may also occur when using proxies based on milk fatty acid profiles since 3-nitrooxypropanol substantially reduces the methane yield but has little or no effect on the fatty acid profile of milk [75]. Another limitation of our equations is that the minimum methane yield it can predict is given by the value of the constant term. This is especially problematic if methane yield is to be predicted for diets that contain halogenated methane analogues (e.g., red seaweed) that may depress methane yield to a value approaching zero [73,76].

Further work is necessary to better understand the relationships between methane yield and ruminal VFA. More frequent VFA determination, or daily VFA proportions collected in breath, may be one way of improving the relationships. The breath collection apparatus as described by Deighton et al. [33] has been shown to be able to collect a sample that represents the entire day’s output from breathing and eructation. Using breath samples would require a reconstruction of the prediction equation since reported proportions of VFA in breath [77] are quite different to those we observed in ruminal fluid.

Potential applications of the equations that we have presented include: (1) They could be used to predict methane yields of individuals or groups of grazing cattle, especially under extensive grazing situations; (2) the equations could be used under confinement feeding situations such as in TMR dairies and in beef feedlots to estimate the methane yields of a representative sub-group of animals. In these circumstances, the total feed intake of a group of animals is usually known; therefore, when estimated methane yields are combined with group intake, the equations allow the estimation of group methane emissions. This approach is likely to be the cheapest and most practical way to estimate methane emissions in these situations. (3) Our equations could be used in research to estimate methane yield from VFA data previously published in many scientific articles, and then meta-analyses could be conducted to test many theories. (4) Lastly, our equations could be used in large-scale screening programs to identify low methane emitting animals.

## 5. Conclusions

VFA proportions in ruminal fluid can be used to predict methane yields of individual or groups of dairy or beef cattle.

## Figures and Tables

**Table 1:** Abbreviations, their descriptions and units for variables and parameters used in the equations.

Abbreviation	Description	Units
A	acetate proportion	mol/100 mol total VFA
B	total butyrate proportion	mol/100 mol total VFA
C2	acetate quantity	mol
C3	propionate quantity	mol
C4	total butyrate quantity	mol
CH_4_P	methane production, stoichiometry	mol
CH_4_Y	methane yield, stoichiometry	mol/100 mol total VFA
DMI	dry matter intake	kg/d
MY	methane yield, predicted	g methane/kg DMI
P	propionate proportion	mol/100 mol total VFA
[P]	propionate concentration	mmol/L
VFA	Volatile fatty acids	mol

**Table 2:** Details of the seven experiments and means for 24 treatments from which individual cow data were used to develop the prediction equations.

Expt	Reference	Expt Design ^1^	Dietary Treatment	n Cows	Methane Method	DIM	Diet Base	Season	Milk Yield	ECM ^2^	Forage DMI	Concentrate DMI	Total DMI
									(kg/Day)	(kg/Day)	(kg/Day)	(kg/Day)	(kg/Day)
1	[24]	Randomized block	Control (lucerne)	12	SF_6_	179	Lucerne	Autumn	25.6	26.4	14.2	8.2	22.4
			Almond hulls	10		177			23.2	24.6	14.4	8.2	22.6
			Citrus pulp	10		176			25.9	25.4	13.2	7.8	21.0
2	[25]	Randomized block	DHA-0 g	8	Calorimeter	218	Lucerne	Autumn	22.2	25.4	18.2	5.9	24.1
			DHA-25 g	7		215			26.0	24.0	18.0	6.1	24.1
			DHA-50 g	8		216			23.1	21.9	16.5	6.2	22.7
			DHA-75 g	7		215			22.3	21.6	15.3	6.2	21.5
3	[26]	Full crossover	Control (water)	8	Calorimeter	90	Lucerne	Spring	32.3	32.3	19.1	5.8	24.9
			Fat	7		95			34.5	32.0	17.8	5.9	23.7
			Fat Plus Tannin	8		90			33.7	31.3	17.9	5.9	23.8
			Tannin	8		90			31.1	30.0	18.7	5.9	24.6
4	[27]	Randomized block	Control (lucerne)	12	SF_6_	149	Lucerne	Summer	23.9	22.7	15.4	5.4	20.8
			Forage brassica	10		150			27.5	25.4	15.2	5.4	20.6
			Chicory	10		145			20.4	19.3	12.3	5.4	17.7
5	[28]	Full crossover	Corn	14	Calorimeter	191	Lucerne	Autumn	27.4	28.6	10.1	12.2	22.3
			Wheat	14		191			29.1	23.8	9.0	11.5	20.5
6	[29]	Randomized block	Wheat-0 kg	8	SF_6_	57	Pasture	Spring	29.9	29.5	17.1	2.1	19.2
			Wheat-3 kg	8		57			31.3	32.4	15.4	5.0	20.4
			Wheat-6 kg	8		57			32.3	33.0	12.3	7.9	20.2
			Wheat-9 kg	8		57			34.7	32.9	8.9	10.9	19.8
7	[30]	Randomized block	Corn 2 times/d	8	SF_6_	206	Lucerne	Autumn	21.2	20.6	8.7	10.2	18.9
			Wheat 2 times/d	8		157			21.3	22.0	8.8	10.2	19.0
			Wheat 6 times/d	8		161			24.0	22.6	8.7	10.1	18.8
			Wheat + buffer, 2 times/d	8		197			20.5	22.4	8.8	10.4	19.2

^1^ Expt = Experiment, DIM = days in milk, DMI = dry matter intake, DHA = docosahexanoic acid, control diet is unique within experiment. ^2^ ECM = energy corrected milk as per Tyrrell and Reid [31].

**Table 3:** Summary of data used to develop models between methane yield and individual volatile fatty acids.

Parameter	Methane Yield (g CH_4_/kg DMI)	Acetate (M%)	Propionate (M%)	Butyrate (M%)
Characteristics				
Mean (±s.d.)	20.7 (4.81)	63.3 (4.53)	20.2 (5.44)	9.9 (4.02)
Minimum	6.9	51.3	12.9	0.7
Maximum	32.4	70.7	40.2	15.6

**Table 4:** Summary of the data used to externally validate the prediction equations.

Experiment	Experiment Design	N ^1^	Animals	Methane Method	Days in Milk	Diet Base	Methane Yield	Acetate	Propionate	Butyrate
							(g/kg DM)	(M%)	(M%)	(M%)
[6]	Randomized block	32	Dairy cows	SF_6_	71	Lucerne hay	10.1–26.9	51.5–67.4	16.3–38.5	6.5–15.3
[14]	Full crossover	3	Dairy cows	Calorimeter	155	TMR, barley silage	18.5–19.2	61.5–61.7	22.8–23.1	11.5–11.5
[36]	Randomized block	4	Beef heifers	Calorimeter	-	TMR, barley	9.2–24.8	42.6–64.0	20.5–45.7	7.1–12.6
[37]	Latin square	4	Beef heifers	Calorimeter	-	Barley silage	19.9–21.6	59.0–65.0	20.6–25.9	9.2–11.4
[38]	Latin square	3	Beef cattle	Calorimeter	-	Barley silage	18.5–18.8	64.6–65.8	19.0–19.6	11.5–11.6
[39]	Crossover	4	Dairy cows	Calorimeter	96	Barley silage and grain	13.4–16.3	59.9–61.7	25.1–26.7	8.7–10.3
[40]	Latin square	4	Dairy cows	Calorimeter	99	Alfalfa and corn silage	18.9–20.6	60.1–63.4	21.8–23.1	12.3–14.4
[41]	Latin square	4	Dairy cows	Calorimeter	61	Grass silage	17.3–21.3	69.5–70.4	15.1–18.6	8.1–11.8
[42]	Latin square	4	Dairy cows	Calorimeter	61	Corn silage	18.7–20.2	64.7–66.1	18.0–19.8	10.4–12.8
[43]	Randomized block	2	Dairy cows	Calorimeter	-	Grass hay	20.7–25.0	67.0–70.2	158–19.4	10.1–10.4
[44]	Latin square	3	Dairy cows	Calorimeter	92	Silage	19.7–20.1	66.6–67.8	19.2–19.5	10.2–11.0
[45]	Randomized block	4	Dairy cows	Calorimeter	215	TMR, grass silage	21.5–22.4	67.9–69.1	15.6–16.8	11.1–11.8
[46]	Randomized block	8	Dairy cows	Calorimeter	139	TMR, corn	19.7–23.1	64.2–66.7	18.0–20.8	11.5–12.4
[47]	Randomized block	2	Beef steers	Calorimeter	-	Grass hay	22.9–23.2	70.0–73.5	16.1–16.6	7.6–10.2
[48]	Latin square	4	Beef steers	Calorimeter	-	TMR, barley silage	18.8–22.6	64.0–68.0	18.3–21.6	8.6–10.4
[49]	Crossover	8	Dairy cows	Calorimeter	187	Grass clover silage	15.4–23.4	60.2–69.7	15.4–26.5	9.3–13.5
[50]	Randomized block	4	Dairy cows	Calorimeter	192	Grass or corn silage	22.0–25.0	63.6–66.0	17.1–18.9	12.8–16.3
[51]	Randomized block	2	Dairy cows	Calorimeter	176	TMR, grass silage	20.5–22.1	58.6–60.3	19.8–23.0	13.6–15.3

^1^ n is number of animals for [6] and number of treatments for all other sources.

**Table 5:** Seven models, used to predict methane yield (MY, g methane/kg dry matter intake) from ruminal acetate (A), propionate (P), and butyrate (B) expressed as mol/100 mol total volatile fatty acid, and the concentration of propionate in ruminal fluid ([P], mmol/L)**.** Their coefficients ^1^ and constants ^2^ were estimated by fitting the models to training data, pooled from seven experiments by meta-analysis. Root-mean-square error of prediction (RMSEP, g CH_4_/kg dry matter intake) and Lin’s concordance (CCC) were calculated by a leave-one-experiment-out cross-validation procedure. These were used to calculate rank (1 = minimum RMSEP, maximum CCC) of equations tested.

Equation Number	Model	Estimates ± S.E.	RMSEP	CCC	Rank
(1c)	MY = 16 × (0.50A − 0.25P + 0.50B) × c/100	c = 3.98 ± 0.15	3.7	0.52	4
(2)	MY = dA − eP + fB	d = 0.30 ± 0.042, e = 0.22 ± 0.055, f = 0.48 ± 0.16	3.6	0.63	4
(3)	MY = g(A/P) + h	g = 4.08 ± 0.36, h = 7.05 ± 1.40	3.2	0.69	1
(4)	MY = i(A+B)/P + j	i = 3.28 ± 0.29. j = 7.60 ± 1.28	3.2	0.70	1
(5)	MY = kP + m	k =−0.57 ± 0.057, m = 32.3 ± 1.4	3.5	0.63	4
(6)	MY = n[P] + q	n =−0.24 ± 0.035, q = 26.1 ± 1.3	4.2	0.36	7
(7)	MY = s/P + t	s = 316 ± 28, t = 4.4 ± 1.5	3.2	0.70	1

^1^ Coefficient c has units of moles of total VFA/kg DMI, coefficients d, e, f, k, and q have units of g CH_4_·kg DMI^−1^·moles^−1^·100 moles total VFA, the coefficients g, and i have units of g CH_4_·kg DMI^−1^ and the coefficient m has units of g CH_4_·kg DMI^−1^·L·mmol^−1^. ^2^ Constants h, j, l, n, and s have the units of g CH_4_·kg DMI^−1^.
